# Molecular Structure and Phylogenetic Analyses of Complete Chloroplast Genomes of Two *Aristolochia* Medicinal Species

**DOI:** 10.3390/ijms18091839

**Published:** 2017-08-24

**Authors:** Jianguo Zhou, Xinlian Chen, Yingxian Cui, Wei Sun, Yonghua Li, Yu Wang, Jingyuan Song, Hui Yao

**Affiliations:** 1 Key Lab of Chinese Medicine Resources Conservation, State Administration of Traditional Chinese Medicine of the People’s Republic of China, Institute of Medicinal Plant Development, Chinese Academy of Medical Sciences & Peking Union Medical College, Beijing 100193, China; jgzhou1316@163.com (J.Z.); chenxinlian1053@163.com (X.C.); yxcui2017@163.com (Y.C.); ywang@implad.ac.cn (Y.W.); jysong@implad.ac.cn (J.S.); 2 Institute of Chinese Materia Medica, China Academy of Chinese Medicinal Sciences, Beijing 100700, China; wsun@icmm.ac.cn; 3 Department of Pharmacy, Guangxi Traditional Chinese Medicine University, Nanning 530200, China; liyonghua185@126.com

**Keywords:** *Aristolochia debilis*, *Aristolochia contorta*, chloroplast genome, molecular structure, phylogenetic analyses

## Abstract

The family Aristolochiaceae, comprising about 600 species of eight genera, is a unique plant family containing aristolochic acids (AAs). The complete chloroplast genome sequences of *Aristolochia debilis* and *Aristolochia contorta* are reported here. The results show that the complete chloroplast genomes of *A. debilis* and *A. contorta* comprise circular 159,793 and 160,576 bp-long molecules, respectively and have typical quadripartite structures. The GC contents of both species were 38.3% each. A total of 131 genes were identified in each genome including 85 protein-coding genes, 37 tRNA genes, eight rRNA genes and one pseudogene (*ycf1*). The simple-sequence repeat sequences mainly comprise A/T mononucletide repeats. Phylogenetic analyses using maximum parsimony (MP) revealed that *A. debilis* and *A. contorta* had a close phylogenetic relationship with species of the family Piperaceae, as well as Laurales and Magnoliales. The data obtained in this study will be beneficial for further investigations on *A. debilis* and *A. contorta* from the aspect of evolution, and chloroplast genetic engineering.

## 1. Introduction

The traditional Chinese medicine plants, *Aristolochia debilis* and *Aristolochia contorta*, are herbaceous climbers in the family Aristolochiaceae. *Aristolochiae fructus* originates from the mellow fruit of the two species, while *Aristolochiae herba* originates from their dried aerial parts. *Aristolochiae fructus* and *Aristolochiae herba* have been recorded as traditional herbal medicines which can clear lung-heat to stop coughing and activate meridians to stop pain, respectively [1]. Modern pharmacology studies have shown that the primary chemical constituents of the two species are aristolochic acid analogues including aristolochic acids (AAs) and aristolactams (ALs) [2,3]. AAs and ALs have been found among species from the family Aristolochiaceae [4]. Previous researches have revealed that AAs are able to react with DNA to form covalent dA-aristolactam (dA-AL) and dG-aristolactam (dG-AL) adducts [5,6]. With further research, current evidence from studies of AAs has demonstrated that AAs can cause nephrotoxicity, carcinogenicity, and mutagenicity [7,8,9,10], especially after prolonged low-dose or shortdated high-dose intake [11,12]. Some nephropathy and malignant tumours including renal interstitial fibrosis, Balkan endemic nephropathy, and upper tract urothelial carcinomas are caused by AAs [13,14,15]. Currently, there are different degrees of restrictions on the sale and use of AAs-containing herbal preparations in many countries.

Chloroplasts are key and semi-autonomous organelles for photosynthesis and biosynthesis in plant cells [16,17,18]. The chloroplast genome, one of three major genetic systems (the other two are nuclear and mitochondrial genomes), is a circular molecule with a typical quadripartite structure of 115 to 165 kb in length [19,20]. All chloroplast genomes of land plants, apart from several rare exceptions, are highly conserved in terms of size, structure, gene content, and gene [21,22,23]. Due to its self-replication mechanism and relatively independent evolution, the genetic information from the chloroplast genome has been used in studies of molecular markers, barcoding identification, plant evolution and phylogenetic [24,25,26]. In 1976, Bedbrook and Bogorad produced the first chloroplast physical mapping of *Zea mays* by digestion with multiple restriction enzymes [27]. Subsequently, the first complete chloroplast genome sequence of *Nicotiana tabacum* was determined [28]. With the development of sequencing technology and bioinformatics, research into the chloroplast genome has increased rapidly. By now, the number of chloroplast genome sequence recorded in the National Center for Biotechnology Information (NCBI) has reached more than 1,500 plant species [29].

About eight genera and 600 species are classified within Aristolochiaceae, and are primarily distributed in tropical and subtropical regions. Of these plants, there are four genera (one endemic) and 86 species (69 endemic) distributed widely in China. The genus *Aristolochia L.*, comprising about 400 species (45 species in China), is the largest and most representative genus of Aristolochiaceae [30]. However, there are no reports on the chloroplast genomes of the family Aristolochiaceae at present, and this has hindered our understanding and progress in the research of the evolution, phylogeny, species identification, and genetic engineering of Aristolochiaceae.

In this study, we determined the complete chloroplast genome sequences of *A. debilis* and *A. contorta*, which are the first two sequenced members of the family Aristolochiaceae. Furthermore, to reveal the phylogenetic positions of the two species, we conducted a phylogenetic tree using the maximum parsimony (MP) method based on common protein-coding genes from 37 species. Overall, the results provide basic genetic information on the chloroplast of *A. debilis* and *A. contorta*, and the role of the two species in plant systematics.

## 2. Results and Discussion

### 2.1. The Chloroplast Genome Structures of A. debilis and A. contorta

**Figure 1:**
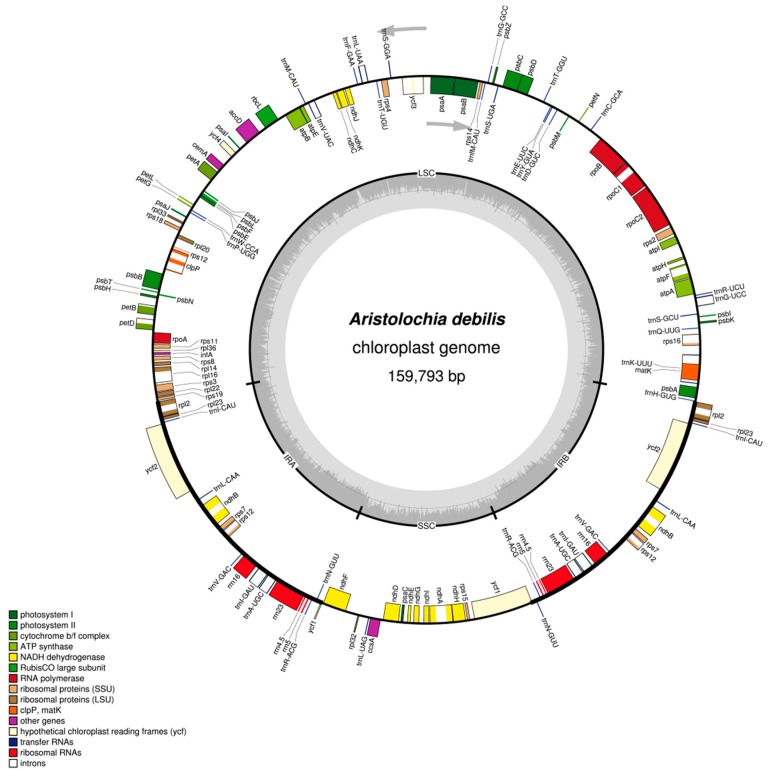
Gene map of the complete chloroplast genome of *A. debilis*. Genes on the inside of the circle are transcribed clockwise, while those outside are transcribed counter clockwise. The darker gray in the inner circle corresponds to GC content, whereas the lighter gray corresponds to AT content.

**Figure 2:**
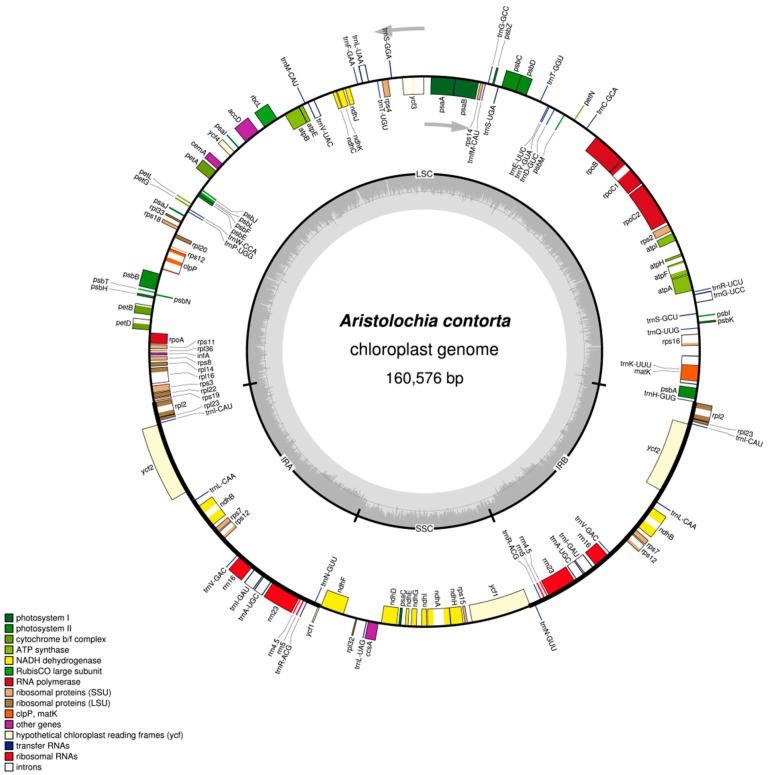
Gene map of the complete chloroplast genome of *A. contorta*. Genes on the inside of the circle are transcribed clockwise, while those outside are transcribed counter clockwise. The darker gray in the inner circle corresponds to GC content, whereas the lighter gray corresponds to AT content.

Both species displayed a typical quadripartite structure, and the corresponding regions were of similar lengths. The complete chloroplast genome of *A. debilis* is a circular molecule of 159,793 bp in length comprising a large single-copy (LSC) region of 89,609 bp and a small single-copy (SSC) region of 19,834 bp separated by a pair of inverted repeats (IRs), each 25,175 bp in length (Figure 1, Table 1). The complete chloroplast genome of *A. contorta* is 160,576 bp in length, which is divided into one LSC (89,781 bp), one SSC (19,877 bp) and two IRs, each 25,459 bp in length (Figure 2, Table 1).

The analysis results revealed that both species had a GC content of 38.3%. However, this was unevenly distributed across the whole chloroplast genome. In both species, the GC content exhibited the highest values of the IR regions across the complete chloroplast genome, 43.4% both in *A. debilis* and *A. contorta*. The high GC content in the IR regions was the result of four rRNA genes (*rrn16*, *rrn23*, *rrn4.5* and *rrn5*) that occur in this region [31]. In addition, the LSC regions have GC contents of 36.6% and 35.5%, as well as the lowest values of 32.9% and 32.8% are seen in SSC regions in *A. debilis* and *A. contorta*, respectively. Within the protein-coding regions (CDS) of chloroplast genome of *A. debilis*, the percentage of AT content for the first, second and third codon positions were 54%, 61.4% and 68%, respectively (Table 1). A bias towards a higher AT representation at the third codon position has also been observed in other land plant chloroplast genomes [32,33,34].

A total of 131 genes were identified from each genome including 85 protein-coding genes, 37 tRNAs, eight rRNAs, and one pseudogene (*ycf1*) (Table 2). The functional *ycf1* copy existed encompassing IR-SSC boundary and the other pseudogene *ycf1* copy was on the other IR region. Six protein-coding genes, seven tRNA genes, and all rRNA genes were duplicated in the IR regions. Coding regions including protein-coding genes (CDS), tRNAs, and rRNAs constituted 56.7% and 56.4% in the chloroplast genomes of *A. debilis* and *A. contorta*, respectively; while the non-coding regions including introns, pseudogenes, and intergenic spacers constituted 43.3% and 43.6% of the genome, respectively.

Introns play an important role in the regulation of gene expression and can enhance the expression of exogenous genes at specific sites and specific times of the plant [35]. The intron content of genes reserved in the chloroplast genomes of *A. debilis* and *A. contorta* are maintained in other angiosperms [31,36]. Data revealed the presence of 18 genes containing introns in each chloroplast genome, including *atpF*, *rpoC1*, *ycf3*, *rps12*, *rpl2*, *rpl16*, *clpP*, *petB*, *petD*, *rps16*, *ndhA*, *ndhB*, and six tRNA genes (Table 3). In addition, the *ycf3* gene and *rps12* gene each contain two introns and three exons. The *ycf3* gene is located in LSC region and the *rps12* gene is a special trans-splicing gene, the 5′ exon is located in LSC, while the 3′ exon is located in IR, which is similar to that in *Aquilaria sinensis* [25], *Panax ginseng* [36] and *Cistanche deserticola* [37].

### 2.2. IR Contraction and Expansion

**Figure 3:**
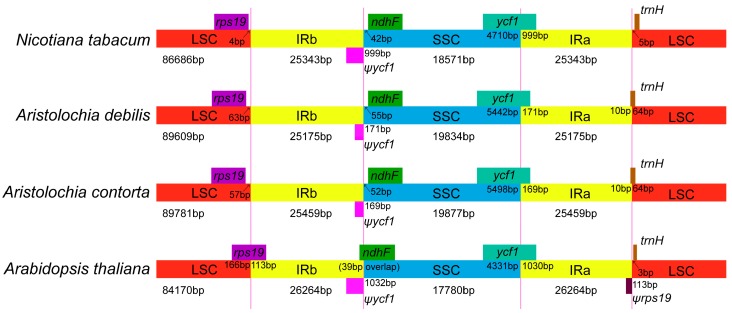
Comparison of the borders of LSC, SSC and IR regions among four chloroplast genomes. Number above the gene features means the distance between the ends of genes and the borders sites. The IRb/SSC border extended intothe *ycf1* genes to create various lengths of *ycf1* pseudogenes among four chloroplast genomes. These features are not to scale.

Although genomic structure and size were highly conserved in Angiosperms chloroplast genomes, the IR/SC boundary regions still varied slightly (Figure 3). The contraction and expansion at the borders of the IR regions are common evolutionary events and represent the main reasons for size variation of the chloroplast genomes [33,38]. From Figure 3, the junctions of the IR and LSC regions of four species including *Arabidopsis thaliana* (accession number: NC_000932), *Nicotiana tabacum* (NC_001879), as well as two *Aristolochia* species were compared. The IRb/SSC border extended into the *ycf1* genes to cause long *ycf1* pseudogenes in all species; however, compared with *A. thaliana* and *N. tabacum*, the length of *ycf1* pseudogene of two *Aristolochia* species were only 171 and 169 bp, respectively. The IRa/SSC border was located in the CDS of the *ycf1* gene and expanded the same length into the 5′ portion of *ycf1* gene as IRb expanded in the four chloroplast genomes. The *trnH* genes were located in the LSC regions in *Nicotiana tabacum*, *Sesamum indicum*, *Arabidopsis thaliana*, and *Salvia miltiorrhiza* [31], while this gene was usually located in the IR region in the monocot chloroplast genomes [39]. Interestingly, the IRa/LSC borders were located in the coding region of *trnH* genes in the two *Aristolochia* species.

### 2.3. Codon Usage and RNA Editing Sites

**Figure 4:**
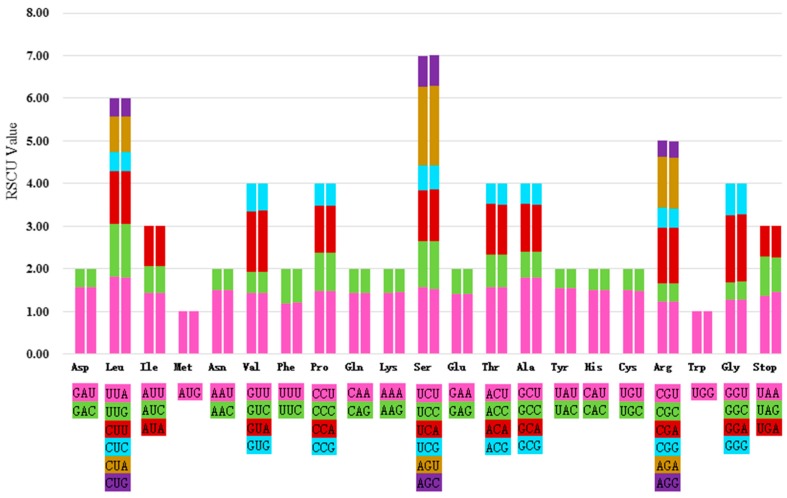
Codon content of 20 amino acid and stop codons in all protein-coding genes of the chloroplast genomes of two *Aristolochia* species. The histogram on the left-hand side of each amino acid shows codon usage within the *A. debilis* chloroplast genome, while the right-hand side illustrates the genome of *A. contorta*.

All the protein-coding genes were composed of 26,239 and 26,255 codons in the chloroplast genomes of *A. debilis* and *A. contorta*, respectively. Among these codons, 2737 encode leucine and 315 encode cysteine, respectively, the most and least universal amino acids in the *A. debilis* chloroplast genome. The codon usages of protein-coding genes in the *A. debilis* and *A. contorta* chloroplast genomes are deduced and summarized in Figure 4 and Table S1. Figure 4 shows that the relative synonymous codon usage (RSCU) value increased with the quantity of codons that code for a specific amino acid. Most of the amino acid codons have preferences except for methionine and tryptophan. The results presented here are similar in codon usage with the chloroplast genomes of species within the genus *Ulmus* [40] and *Aq. sinensis* [25]. In addition, potential RNA editing sites were predicted for 35 genes of the chloroplast genomes of two species. A total of 92 RNA editing sites were identified (Table S2). The amino acid conversion S to L occurred most frequently, while P to S and R to W occurred least. Seventy-six common RNA editing sites were shared in genes of the two species.

### 2.4. Repeat Structure and Simple Sequence Repeats Analyses

**Figure 5:**
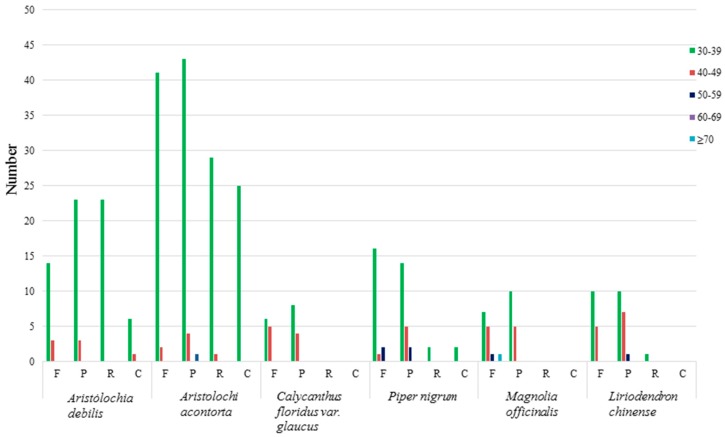
Repeat sequences in six chloroplast genomes. REPuter was used to identify repeat sequences with length ≥30 bp and sequence identified ≥90% in the chloroplast genomes. F, P, R, and C indicate the repeat types F (forward), P (palindrome), R (reverse), and C (complement), respectively. Repeats with different lengths are indicated in different colours.

The repeats were mostly distributed in the intergenic spacer (IGS) and intron sequences. Figure 5 shows the repeat structure analyses of six species. The results revealed that the repeats of chloroplast genome of *A. contorta* had the greatest number, comprising of 41 forward, 43 palindromic, 29 reverse, and 25 complement repeats. Followed by *A. debilis*, contained 14 forward, 23 palindromic, 23 reverse, and six complement repeats. Simple sequence repeats (SSRs), which are ubiquitous throughout the genomes and are also known as microsatellites, are tandemly repeated DNA sequences that consist of 1–6 nucleotide repeat units [41]. SSRs are widely used for molecular markers in species identification, population genetics, and phylogenetic investigations based on their high level of polymorphism [42,43,44]. A total of 129 and 156 SSRs were identified using the microsatellite identification tool (MISA) in the chloroplast genomes of *A. debilis* and *A. contorta*, respectively (Table 4; Tables S3 and S4). In these SSRs, mononucletide repeats were largest in number, which were found 81 and 96 times in *A. debilis* and *A. contorta*, respectively. A/T mononucleotide repeats (96.3% and 94.8%, respectively) were the most common, while the majority of dinucleotide repeat sequences comprised of AT/TA repeats (100% and 92.8%, respectively). This result agreed with the previous studies where proportions of polyadenine (polyA) and polythymine (polyT) were higher than polycytosine (polyC) and polyguanine (polyG) within chloroplast SSRs in many plants [24].

### 2.5. Comparative Genomic Analysis

**Figure 6:**
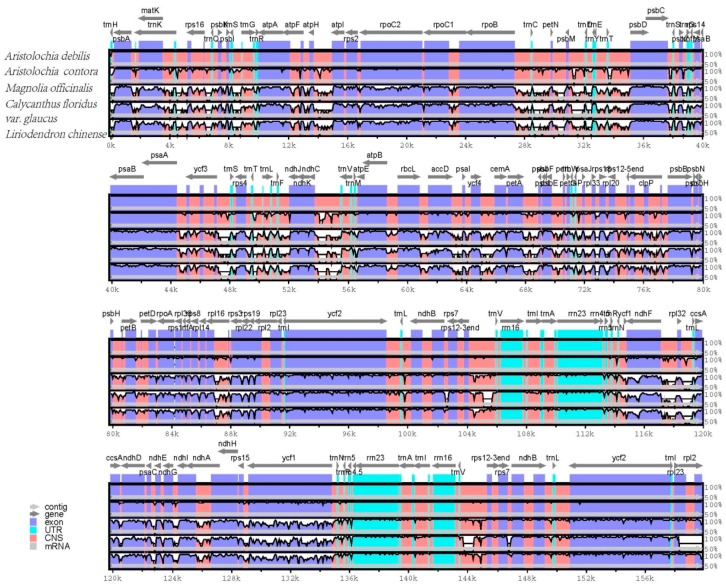
Sequence identity plot comparing the five chloroplast genomes with *A. debilis* as a reference by using mVISTA. Grey arrows and thick black lines above the alignment indicate genes with their orientation and the position of the IRs, respectively. A cut-off of 70% identity was used for the plots, and the Y-scale represents the percent identity ranging from 50% to 100%.

The whole chloroplast genome sequences of *A. debilis* and *A. contorta* were compared to those of *Calycanthus floridus* var. *glaucus* (accession number: NC_004993), *Magnolia officinalis* (NC_020316), and *Liriodendron chinense* (NC_030504) using the mVISTA program (Figure 6). The comparison showed that the two IR regions were less divergent than the LSC and SSC regions. The four rRNA genes were the most conserved, while the most divergent coding regions were *ndhF*, *rpl22*, *ycf1*, *rpoC2* and *ccsA*. Additionally, the results revealed that non-coding regions exhibited a higher divergence than coding regions, and the most divergent regions localized in the intergenic spacers among the five chloroplast genomes.

### 2.6. Phylogenetic Analyses

**Figure 7:**
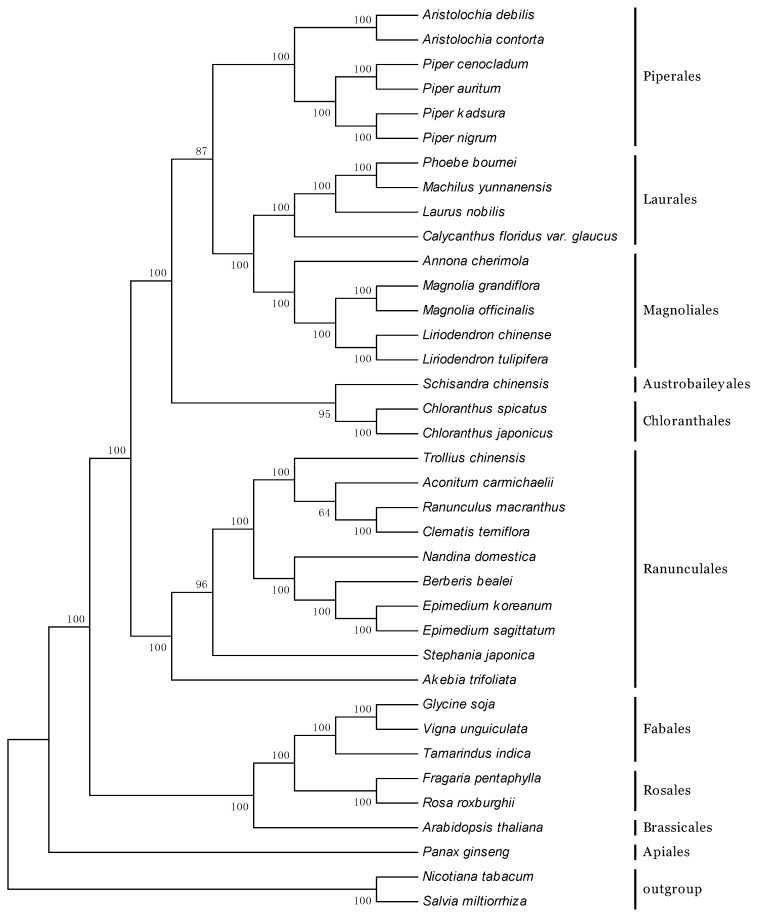
Phylogenetic tree constructed using Maximum parsimony (MP) method based on 60 protein-coding genes from different species. Numbers at nodes are values for bootstrap support.

Chloroplast genomes provide abundant resources, which are significant for evolutionary, taxonomic, and phylogenetic studies [31,45,46]. The whole chloroplast genomes and protein-coding genes have been successfully used to resolve phylogenetic relationships at almost any taxonomic level during the past decade [31,37]. *Aristolochia*, consisting of nearly 400 species, is the largest genus in the family Aristolochiaceae [30]. Phylogenetic analyses employing one or several genes have been performed in previous studies [47,48,49]; however, these analyses were restricted to the species of Aristolochiaceae and included few species from other families. In this study, to identify the phylogenetic positions of *A. debilis* and *A. contorta* within Angiosperms, 60 protein-coding genes commonly present in 37 species from Piperales, Laurales, Magnoliales, Ranunculales, Fabales, Rosales, Chloranthales, as well as two *Aristolochia* species were used to construct the phylogenetic tree using the Maximum parsimony (MP) method (Figure 7). All the nodes in the MP trees have high bootstrap support values, and 30 out of 34 nodes with 100% bootstrap values were found. The result illustrated that two *Aristolochia* species were sister taxa with respect to four *Piper* species (Piperaceae), and these species were grouped with four species from Laurales and five species from Magnoliales. Additionally, all species are clustered within a lineage distinct from the outgroup. This result (inferred from the chloroplast genome data) obtained high support values, which suggested that the chloroplast genome could effectively resolve the phylogenetic positions and relationships of this family. Nevertheless, to accurately illustrate the evolution of the family Aristolochiaceae, it is necessary to use more species to analyze the phylogeny. This study will also provide a reference for species identification among *Aristolochia* and other genus using the chloroplast genome.

## 3. Materials and Methods

### 3.1. Plant Material, DNA Extraction, and Sequencing

Fresh plants of *A. debilis* and *A. contorta* were collected from Lichuan City in Hubei Province and Tonghua City in Jilin Province, respectively. All samples were identified by Professor Yulin Lin, who is based at the Institute of Medicinal Plant Development (IMPLAD), Chinese Academy of Medical Sciences (CAMS) and Peking Union Medical College (PUMC). The voucher specimens were deposited in the herbarium of the IMPLAD. The leaves were cleansed and preserved in a −80 °C refrigerator. Total genomic DNA was extracted from approximately 100 mg of samples using DNeasy Plant Mini Kit with a standard protocol (Qiagen Co., Hilden, Germany). Final DNA quality was assessed based on spectrophotometry and their integrity was examined by electrophoresis in 1% (*w*/*v*) agarose gel. The DNA was used to construct shotgun libraries with insert sizes of 500 bp and sequenced according to the manufacturer’s manual for the Illumina Hiseq X. Approximately 6.3 Gb of raw data from *A. debilis* and 5.8 Gb from *A. contorta* were produced with 150 bp pair-end read lengths.

### 3.2. Chloroplast Genome Assembly and Annotation

First, we used the software Trimmomatic (v0.36, Max Planck Institute of Molecular Plant Physiology, Potsdam, Germany) [50] to trim the low-quality reads. After quality control, the clean reads were used to assemble the chloroplast genome. All chloroplast genomes of plants recorded in the National Center for Biotechnology Information (NCBI) were used to construct a reference database. Next, the clean reads were mapped to the database on the basis of their coverage and similarity, and the mapped reads extracted. Extracted reads were assembled to contigs using SOAPdenovo (v2, BGI HK Research Institute, Hong Kong, China) [51], and the resulting contigs were combined and extended to obtain a complete chloroplast genome sequence. To verify the accuracy of assembly, four boundaries of single copy (SC) and inverted repeat (IR) regions of the assembled sequences were confirmed by PCR amplification and Sanger sequencing using the primers listed in Table S5.

We used the online program Dual Organellar GenoMe Annotator (DOGMA), (University of Texas at Austin, Austin, TX, USA) [52] and the software Chloroplast Genome Annotation, Visualization, Analysis, and GenBank Submission (CPGAVAS), (Institute of Medicinal Plant Development, Chinese Academy of Medical Sciences and Peking Union Medical College, Beijing, China) [53] coupled with manual corrections to perform the preliminarily gene annotation of chloroplast genomes of two species. The tRNA genes were identified using the software tRNAscan-SE (v2.0, University of California, Santa Cruz, CA, USA) [54] and DOGMA [52]. The gene map was drawn using the Organellar Genome DRAW (OGDRAW) (v1.2, Max Planck Institute of Molecular Plant Physiology, Potsdam, Germany) [55] with default settings and checked manually. The complete and correct chloroplast genome sequences of the two species were deposited in GenBank, accession numbers of *A. debilis* and *A. contorta* are MF539928 and MF539927, respectively.

### 3.3. Genome Structure Analyses and Genome Comparison

The distribution of codon usage was investigated using the software CodonW ( University of Texas, Houston, TX, USA) with the RSCU ratio [56]. Thirty-five protein-coding genes of the chloroplast genomes of two species were used to predict potential RNA editing sites using the online program Predictive RNA Editor for Plants (PREP) suite [57] with a cutoff value of 0.8. GC content was analyzed using Molecular Evolutionary Genetics Analysis (MEGA v6.0, Tokyo Metropolitan University, Tokyo, Japan) [58]. REPuter ( University of Bielefeld, Bielefeld, Germany) [59] to identify the size and location of repeat sequences, including forward, palindromic, reverse, and complement repeats in the chloroplast genomes of six species *C. floridus* var. *glaucus*, *M. officinalis*, *L. chinense* and *Piper nigrum* (NC_034692). For all repeat types, the minimal size was 30 bp and the two repeat copies had at least 90% similarity. Simple sequence repeats (SSRs) were detected using MISA software [60] with parameters set the same as Li et al. [61]. The whole-genome alignment for the chloroplast genomes of the five species including *A. debilis*, *A. contorta*, *C. floridus* var. *glaucus*, *M. officinalis*, and *L. chinense* were performed and plotted using the mVISTA program [62].

### 3.4. Phylogenetic Analyses

A total of 35 complete chloroplast genomes were downloaded from the NCBI Organelle Genome Resources database (Table S6). The 60 protein-coding gene sequences commonly present in 37 species, including the two species in this study, were aligned using the Clustal algorithm [63]. To determine the phylogenetic positions of *A. debilis*, and *A. contorta*, we analyzed the chloroplast genomes of these 60 protein-coding genes. Maximum parsimony (MP) analysis was performed with PAUP*4.0b10 [64], using a heuristic search performed with the MULPARS option, the random stepwise addition with 1000 replications and tree bisection-reconnection (TBR) branch swapping. Bootstrap analysis was also performed with 1,000 replicates with TBR branch swapping.

## 4. Conclusions

The complete chloroplast genomes of *A. debilis* and *A. contorta*, the first two sequenced members of the family Aristolochiaceae, were determined in this study. The genome structure and gene content were relatively conserved. The phylogenetic analyses illustrated that these two *Aristolochia* species were positioned close to four species from the family Piperaceae and had a close phylogenetic relationship with Laurales and Magnoliales. The results provided the basis for the study of the evolutionary history of *A. debilis* and *A. contorta*. All the data presented in this paper will facilitate the further investigation of these two medicinal plants.

## Figures and Tables

**Table 1:** Base composition in the chloroplast genomes of *A. debilis* and *A. contorta*.

Species	Regions	Positions	T(U) (%)	C (%)	A (%)	G (%)	Length (bp)
*A. debilis*	LSC	-	32.2	18.7	31.2	17.9	89,609
	SSC	-	34.0	17.4	33.2	15.5	19,834
	IRa	-	28.4	22.4	28.3	21.0	25,175
	IRb	-	28.3	21.0	28.4	22.4	25,175
	Total	-	31.2	19.5	30.5	18.8	159,793
	CDS	-	30.9	18.1	30.2	20.8	78,717
	-	1st position	23.5	18.8	30.5	27.2	26,239
	-	2nd position	32.2	20.5	29.2	18.1	26,239
	-	3rd position	36.9	15.1	31.1	17.0	26,239
*A. contorta*	LSC	-	32.2	18.7	31.2	17.8	89,781
	SSC	-	33.9	17.4	33.3	15.4	19,877
	IRa	-	28.4	22.4	28.2	21.0	25,459
	IRb	-	28.2	21.0	28.4	22.4	25,459
	Total	-	31.2	19.5	30.6	18.8	160,576
	CDS	-	30.9	18.1	30.3	20.7	78,765
	-	1st position	23.5	18.8	30.5	27.2	26,255
	-	2nd position	32.2	20.6	29.2	18.1	26,255
	-	3rd position	37.0	15.0	31.1	16.9	26,255

* CDS: protein-coding regions.

**Table 2:** Gene contents in the chloroplast genomes of *A. debilis* and *A. contorta*.

No.	Group of Genes	Gene names	Amount
1	Photosystem I	*psaA*, *psaB*, *psaC*, *psaI*, *psaJ*	5
2	Photosystem II	*psbA*, *psbB*, *psbC*, *psbD*, *psbE*, *psbF*, *psbH*, *psbI*, *psbJ*, *psbK*, *psbL*, *psbM*, *psbN*, *psbT*, *psbZ*	15
3	Cytochrome b/f complex	*petA*, *petB* *, *petD* *, *petG*, *petL*, *petN*	6
4	ATP synthase	*atpA*, *atpB*, *atpE*, *atpF* *, *atpH*, *atpI*	6
5	NADH dehydrogenase	*ndhA* *, *ndhB* *(×2)^1^, *ndhC*, *ndhD*, *ndhE*, *ndhF*, *ndhG*, *ndhH*, *ndhI*, *ndhJ*, *ndhK*	12(1)
6	RubisCO large subunit	*rbcL*	1
7	RNA polymerase	*rpoA*, *rpoB*, *rpoC1* *, *rpoC2*	4
8	Ribosomal proteins (SSU)	*rps2*, *rps3*, *rps4*, *rps7*(×2), *rps8*, *rps11*, *rps12* **(×2), *rps14*, *rps15*, rps16 *, *rps18*, *rps19*	14(2)
9	Ribosomal proteins (LSU)	*rpl2* *(×2), *rpl14*, *rpl16* *, *rpl20*, *rpl22*, *rpl23*(×2), *rpl32*, *rpl33*, *rpl36*	11(2)
10	Proteins of unknown function	*ycf1*, *ycf2*(×2), *ycf3* **, *ycf4*	5(1)
11	Transfer RNAs	37 *tRNA*s (6 contain an intron, 7 in the IRs)	37(7)
12	Ribosomal RNAs	*rrn4.5*(×2), *rrn5*(×2), *rrn16*(×2), *rrn23*(×2)	8(4)
13	Other genes	*accD*, *clpP* **, *matK*, *ccsA*, *cemA*, *infA*	6

* Gene contains one intron; ** gene contains two introns; (×2) indicates the number of the repeat unit is 2.

**Table 3:** Genes with introns in the chloroplast genomes of *A. debilis* and *A. contorta* as well as the lengths of the exons and introns.

Species	Gene	Location	Exon I (bp)	Intron I (bp)	Exon II (bp)	Intron II (bp)	Exon III (bp)
*A. debilis*	*atpF*	LSC	145	805	410	-	-
	*clpP*	LSC	71	781	292	678	255
	*ndhA*	SSC	552	1090	540	-	-
	*ndhB*	IR	777	705	756	-	-
	*petB*	LSC	6	214	642	-	-
	*petD*	LSC	6	485	476	-	-
	*rpl16*	LSC	8	1065	403	-	-
	*rpl2*	IR	391	657	431	-	-
	*rpoC1*	LSC	430	776	1622	-	-
	*rps12*	LSC	114	-	232	536	26
	*rps16*	LSC	46	853	191	-	-
	*trnA-UGC*	IR	38	809	35	-	-
	*trnG-UCC*	LSC	24	761	48	-	-
	*trnI-GAU*	IR	37	937	35	-	-
	*trnK-UUU*	LSC	37	2658	35	-	-
	*trnL-UAA*	LSC	35	521	50	-	-
	*trnV-UAC*	LSC	39	597	37	-	-
	*ycf3*	LSC	126	777	228	753	147
*A. contorta*	*atpF*	LSC	145	771	410	-	-
	*clpP*	LSC	71	821	292	664	255
	*ndhA*	SSC	552	1091	540	-	-
	*ndhB*	IR	777	716	756	-	-
	*petB*	LSC	6	214	642	-	-
	*petD*	LSC	7	485	476	-	-
	*rpl16*	LSC	8	1088	403	-	-
	*rpl2*	IR	391	657	431	-	-
	*rpoC1*	LSC	430	776	1619	-	-
	*rps12*	LSC	114	-	232	536	26
	*rps16*	LSC	46	832	221	-	-
	*trnA-UGC*	IR	38	809	35	-	-
	*trnG-UCC*	LSC	24	751	48	-	-
	*trnI-GAU*	IR	37	938	35	-	-
	*trnK-UUU*	LSC	37	2648	35	-	-
	*trnL-UAA*	LSC	35	552	50	-	-
	*trnV-UAC*	LSC	39	605	37	-	-
	*ycf3*	LSC	126	764	228	760	147

**Table 4:** Types and amounts of SSRs in the *A. debilis* and *A. contorta* chloroplast genomes.

SSR Type	Repeat Unit	Amount		Ratio (%)	
		*A. debilis*	*A. contorta*	*A. debilis*	*A. contorta*
Mono	A/T	78	91	96.3	94.8
	C/G	3	5	3.7	5.2
Di	AC/GT	0	1	0	3.6
	AG/CT	0	1	0	3.6
	AT/TA	19	26	100	92.8
Tri	AAC/GTT	1	1	10	8.3
	AAG/CTT	1	1	10	8.3
	ATC/ATG	1	0	10	0
	AAT/ATT	7	10	70	83.4
Tetra	AAAC/GTTT	2	2	16.7	14.3
	AAAT/ATTT	4	5	33.3	35.7
	AATC/ATTG	1	1	8.3	7.1
	AGAT/ATCT	2	1	16.7	7.1
	AATT/AATT	0	1	0	7.1
	ACAT/ATGT	0	1	0	7.1
	AACT/AGTT	1	1	8.3	7.1
	AATG/ATTC	2	2	16.7	14.3
Penta	AATAT/ATATT	2	2	33.3	50
	AAATT/AATTT	1	0	16.7	0
	AAATC/ATTTG	1	0	16.7	0
	AACAT/ATGTT	0	1	0	25
	AAAAT/ATTTT	2	1	33.3	25
Hexa	AAATAG/ATTTCT	0	1	0	50
	ACATAT/ATATGT	0	1	0	50
	ACTGAT/AGTATC	1	0	100	0

## Supplementary Materials

Supplementary materials can be found at www.mdpi.com/1422-0067/18/9/1839/s1.

## Abbreviations

LSC	Large single copy
SSC	Small single copy
IR	Inverted repeat
MP	Maximum parsimony
SSR	Simple sequence repeats
ATP	Adenosine triphosphate
NADH	Nicotinamide adenine dinucleotide
