# Supplementary material for: Molecular Structure and Phylogenetic Analyses of Complete Chloroplast Genomes of Two Aristolochia Medicinal Species
Source: Int J Mol Sci. 2017 Aug 24;18(9):1839. doi: 10.3390/ijms18091839 (PMC5618488; doi:10.3390/ijms18091839)
Supplement: Supplementary file 1 [file ijms-18-01839-s001.pdf]

Article

Molecular Structure and Phylogenetic Analyses of Complete Chloroplast Genomes of Two *Aristolochia* Medicinal Species

Jianguo Zhou[1], Xinlian Chen[1], Yingxian Cui[1], Wei Sun[2], Yonghua Li[3], Yu Wang[1], Jingyuan Song[1] and Hui Yao[1,*]

[1] Key Lab of Chinese Medicine Resources Conservation, State Administration of Traditional Chinese Medicine of the People's Republic of China, Institute of Medicinal Plant Development, Chinese Academy of Medical Sciences & Peking Union Medical College, Beijing, 100193, China. jgzhou1316@163.com (J.Z.); chenxinlian1053@163.com (X.C.); yxcui2017@163.com (Y.C.); ywang@implad.ac.cn (Y.W.); jysong@implad.ac.cn (J.S.)

[2] Institute of Chinese Materia Medica, China Academy of Chinese Medicinal Sciences, Beijing 100700, China. wsun@icmm.ac.cn (W.S.)

[3] Department of Pharmacy, Guangxi Traditional Chinese Medicine University, Nanning, 530200, Guangxi, China. liyonghua185@126.com (Y. L.)

* Correspondence: scauyaoh@sina.com; Tel: +86-10-57833194.

Supplementary Materials

Table S1 Codon usage within the chloroplast genomes of *A. debilis* and *A. contorta*.

Amino acid	Codon	No.		RSCU		tRNA	Amino acid	Codon	No.		RSCU		tRNA
		①*	②*	①	②				①	②	①	②	
Phe	UUU	869	883	1.2	1.22		Tyr	UAU	736	736	1.55	1.55	
Phe	UUC	577	564	0.8	0.78	*trnF-GAA*	Tyr	UAC	214	211	0.45	0.45	*trnY-GUA*
Leu	UUA	826	821	1.81	1.8	*trnL-UAA*	Stop	UAA	39	41	1.38	1.45	
Leu	UUG	568	570	1.25	1.25	*trnL-CAA*	Stop	UAG	26	23	0.92	0.81	
Leu	CUU	563	562	1.23	1.23		His	CAU	489	487	1.5	1.51	
Leu	CUC	210	214	0.46	0.47		His	CAC	162	160	0.5	0.49	*trnH-GUG*
Leu	CUA	378	376	0.83	0.83	*trnL-UAG*	Gln	CAA	664	669	1.44	1.44	*trnQ-UUG*
Leu	CUG	192	190	0.42	0.42		Gln	CAG	256	260	0.56	0.56	
Ile	AUU	1061	1067	1.44	1.44		Asn	AAU	921	926	1.51	1.51	
Ile	AUC	465	465	0.63	0.63	*trnI-GAU*	Asn	AAC	298	302	0.49	0.49	*trnN-GUU*
Ile	AUA	687	689	0.93	0.93	*trnI-CAU*	Lys	AAA	987	995	1.44	1.45	*trnK-UUU*
Met	AUG	628	622	1	1	*trn(f)M-CAU*	Lys	AAG	383	376	0.56	0.55	
Val	GUU	508	510	1.43	1.44		Asp	GAU	874	874	1.58	1.58	
Val	GUC	178	173	0.5	0.49	*trnV-GAC*	Asp	GAC	235	231	0.42	0.42	*trnD-GUC*
Val	GUA	505	506	1.42	1.43	*trnV-UAC*	Glu	GAA	972	969	1.42	1.42	*trnE-UUC*
Val	GUG	227	230	0.64	0.65		Glu	GAG	393	395	0.58	0.58	
Ser	UCU	534	529	1.56	1.53		Cys	UGU	236	234	1.5	1.49	
Ser	UCC	370	384	1.08	1.11	*trnS-GGA*	Cys	UGC	79	81	0.5	0.51	*trnC-GCA*
Ser	UCA	417	421	1.21	1.22	*trnS-UGA*	Stop	UGA	20	21	0.71	0.74	
Ser	UCG	196	193	0.57	0.56		Trp	UGG	456	459	1	1	*trnW-CCA*
Pro	CCU	407	409	1.48	1.48		Arg	CGU	330	328	1.23	1.23	*trnR-ACG*
Pro	CCC	247	245	0.9	0.89		Arg	CGC	117	118	0.44	0.44	
Pro	CCA	305	309	1.11	1.12	*trnP-UGG*	Arg	CGA	347	347	1.3	1.3	
Pro	CCG	140	139	0.51	0.5		Arg	CGG	126	120	0.47	0.45	
Thr	ACU	528	528	1.56	1.57		Arg	AGA	410	412	1.19	1.19	*trnR-UCU*
Thr	ACC	260	260	0.77	0.77	*trnT-GGU*	Arg	AGG	133	131	0.39	0.38	
Thr	ACA	402	393	1.19	1.17	*trnT-UGU*	Ser	AGU	497	498	1.86	1.87	
Thr	ACG	160	161	0.47	0.48		Ser	AGC	188	191	0.7	0.72	*trnS-GCU*
Ala	GCU	637	640	1.79	1.79		Gly	GGU	583	581	1.28	1.28	
Ala	GCC	221	220	0.62	0.62		Gly	GGC	187	190	0.41	0.42	*trnG-GCC*
Ala	GCA	394	393	1.11	1.1	*trnA-UGC*	Gly	GGA	712	716	1.56	1.57	*trnG-UCC*
Ala	GCG	171	174	0.48	0.49		Gly	GGG	338	333	0.74	0.73	

RSCU: Relative Synonymous Codon Usage. ①*A. debilis*; ②*A. contorta*

Table S2 RNA editing predicted in the chloroplast genomes of two *Aristolochia* species by the PREP program

Gene	Nucleotide Position		Codon change	Amino acid change	Score
	Aristolochia debilis	*Aristolochia contorta*			
*acc*D	295	295	CTT-TTT	L-F	1.00
	848	854	TCG-TTG	S-L	0.80
	1211	1217	TCA-TTA	S-L	1.00
	1457	1463	CCT-CTT	P-L	1.00
*atp*A	773	773	TCA-TTA	S-L	1.00
	914	914	TCA-TTA	S-L	1.00
	-	1148	TCA-TTA	S-L	1.00
*atp*B	1190	1190	TCA-TTA	S-L	1.00
*atp*F	92	92	CCA-CTA	P-L	0.86
*atp*I	428	428	CCC-CTC	P-L	1.00
	629	629	TCA-TTA	S-L	1.00
*ccs*A	514	514	CTT-TTT	L-F	0.86
	644	644	ACT-ATT	T-I	0.86
*clp*P	82	82	CAT-TAT	H-Y	1.00
	559	559	CAC-TAC	H-Y	1.00
*mat*K	-	53	TCC-TTC	S-F	1.00
	202	202	CAT-TAT	H-Y	0.86
	-	325	CTC-TTC	L-F	0.86
	439	439	CAT-TAT	H-Y	0.86
	463	463	CAC-TAC	H-Y	1.00
	649	652	CAT-TAT	H-Y	1.00
	1193	1196	TCG-TTG	S-L	0.86
*ndh*A	50	50	TCG-TTG	S-L	1.00
	107	107	CCT-CTT	P-L	1.00
	317	317	GCC-GTC	A-V	1.00
	341	341	TCA-TTA	S-L	1.00
	476	476	TCA-TTA	S-L	1.00
	566	566	TCA-TTA	S-L	1.00
	922	922	CTC-TTC	L-F	1.00

*ndh*B	149	149	TCA-TTA	S-L	1.00
	467	467	CCA-CTA	P-L	1.00
	542	542	ACG-ATG	T-M	1.00
	586	586	CAT-TAT	H-Y	1.00
	611	611	TCA-TTA	S-L	0.80
	830	830	TCA-TTA	S-L	1.00
	836	836	TCA-TTA	S-L	1.00
	1255	1255	CAT-TAT	H-Y	1.00
	1481	1481	CCA-CTA	P-L	1.00
*ndh*D	23	23	TCC-TTC	S-F	0.80
	575	575	TCA-TTA	S-L	1.00
	650	650	TCA-TTA	S-L	1.00
	854	854	TCA-TTA	S-L	1.00
	863	863	CCA-CTA	P-L	1.00
	1160	1160	GCT-GTT	A-V	0.80
	1274	1274	TCA-TTA	S-L	0.80
	1373	1373	ACC-ATC	T-I	1.00
*ndh*F	290	290	TCA-TTA	S-L	1.00
	586	586	CTT-TTT	L-F	0.80
	869	-	ACA-ATA	T-I	1.00
	890	-	ACA-ATA	T-I	1.00
	1001	1001	TCA-TTA	S-L	1.00
	1783	-	CCT-CTT	P-S	1.00
	2014	2014	CTT-TTT	L-F	1.00
	2233	2234	CTT-TTT	L-F	1.00
*ndh*G	155	155	CCA-CTA	P-L	1.00
	314	314	ACA-ATA	T-I	0.80
*pet*B	418	-	CGG-TGG	R-W	1.00
	611	611	CCA-CTA	P-L	1.00
*pet*D	-	-	-	-	-
*pet*G	-	-	-	-	-
*pet*L	-	83	ACA-ATA	T-I	1.00
*psa*B	1919	1919	ACG-ATG	T-M	1.00
*psa*I	80	80	TCT-TTT	S-F	0.86

*psb*B	-	-	-	-	-
*psb*E	-	-	-	-	-
*psb*F	77	77	TCT-TTT	S-F	1.00
*psb*L	-	-	-	-	-
*rpl*2	2	2	ACG-ATG	T-M	1.00
*rpl*20	256	256	CAC-TAC	H-Y	0.86
	308	308	TCA-TTA	S-L	0.86
*rpl*23	-	-	-	-	-
*rpo*A	82	82	CAT-TAT	S-L	0.86
	368	-	TCA-TTA	S-L	1.00
	-	368	TCG-TTG	S-L	1.00
*rpo*B	338	338	TCT-TTT	S-F	1.00
	473	473	TCA-TTA	S-L	0.86
	566	566	TCG-TTG	S-L	1.00
	1877	1877	GCT-GTT	A-V	0.86
	2033	2033	GCA-GTA	A-V	1.00
*rpo*C1	41	41	TCG-TTG	S-L	1.00
	926	926	CCA-CTA	P-L	1.00
*rpo*C2	1594	1594	CCT-CTT	P-F	1.00
	1595	1595	CCT-CTT	P-F	1.00
	2296	2296	CGG-TGG	R-W	1.00
	2725	2725	CCC-CTC	P-S	1.00
	3047	3047	GCC-GTC	A-V	0.86
	3301	3301	CAT-TAT	H-Y	1.00
	3713	3713	TCA-TTA	S-L	0.86
*rps*2	248	248	TCA-TTA	S-L	1.00
*rps*8	169	169	CAT-TAT	H-Y	0.86
	182	182	TCA-TTA	S-L	0.86
*rps*14	80	80	CCC-CTC	P-L	1.00
	149	149	CCG-CTG	P-L	1.00
*rps*16	212	212	TCA-TTA	S-L	0.83
*ycf*3	44	44	TCC-TTC	S-F	1.00
	53	53	GCA-GTA	A-V	1.00
	185	185	ACG-ATG	T-M	1.00

	407	407	TCT-TTT	S-F	1.00

Table S3 Simple sequence repeats (SSRs) in *A. debilis* chloroplast genome.

SSR nr.	SSR type	SSR	size	start	end
1	p1	(T)11	11	99	109
2	p4	(GATA)3	12	1418	1429
3	p2	(AT)5	10	3892	3901
4	p2	(AT)5	10	4494	4503
5	p1	(A)11	11	4627	4637
6	p1	(T)14	14	4728	4741
7	p3	(ATG)4	12	4802	4813
8	p1	(A)11	11	5130	5140
9	p2	(TA)5	10	6452	6461
10	p1	(A)12	12	6737	6748
11	p1	(A)15	15	7485	7499
12	p1	(A)14	14	8231	8244
13	p5	(TATTA)3	15	8349	8363
14	p1	(T)11	11	8650	8660
15	p1	(T)12	12	8682	8693
16	p1	(T)10	10	9092	9101
17	p1	(A)17	17	11618	11634
18	p1	(A)11	11	12814	12824
19	p3	(TTA)6	18	13120	13137
20	p5	(TATAA)3	15	14143	14157
21	p2	(AT)6	12	14207	14218
22	p2	(AT)7	14	14405	14418
23	p1	(T)16	16	14854	14869
24	p1	(T)11	11	14993	15003
25	p2	(AT)5	10	20283	20292
26	p1	(T)11	11	23368	23378
27	p1	(T)12	12	26621	26632
28	p1	(T)10	10	28042	28051
29	p1	(A)15	15	28222	28236
30	p1	(A)11	11	28650	28660
31	p1	(T)12	12	29448	29459
32	p1	(T)12	12	29462	29473
33	p1	(C)13	13	30202	30214
34	p3	(ATA)4	12	30643	30654
35	p1	(A)11	11	30983	30993
36	p1	(T)11	11	31197	31207
37	p1	(A)10	10	31447	31456
38	p1	(A)11	11	31545	31555
39	p2	(TA)8	16	32976	32991
40	p1	(T)14	14	34255	34268
41	p1	(C)11	11	34499	34509
42	p4	(TTTG)3	12	35078	35089

43	p1	(T)13	13	37750	37762
44	p2	(TA)7	14	37814	37827
45	p1	(A)14	14	38298	38311
46	p1	(A)14	14	38806	38819
47	p2	(AT)11	22	38925	38946
48	p2	(TA)6	12	38948	38959
49	p1	(A)10	10	45060	45069
50	p1	(T)10	10	45907	45916
51	p1	(T)11	11	46890	46900
52	p1	(A)10	10	46955	46964
53	p5	(AATCA)3	15	46967	46981
54	p1	(A)13	13	47228	47240
55	p3	(ATA)4	12	49660	49671
56	p3	(ATA)4	12	49717	49728
57	p1	(A)12	12	49906	49917
58	p1	(A)16	16	50367	50382
59	p2	(TA)7	14	50588	50601
60	p1	(T)11	11	50877	50887
61	p4	(TTTG)3	12	51518	51529
62	p2	(AT)6	12	54289	54300
63	p2	(AT)5	10	54346	54355
64	p1	(T)14	14	54550	54563
65	p1	(T)10	10	56369	56378
66	p5	(TTAAT)3	15	60915	60929
67	p3	(CAA)4	12	63531	63542
68	p1	(A)11	11	64047	64057
69	p2	(AT)7	14	64072	64085
70	p1	(T)12	12	65389	65400
71	p1	(A)10	10	65828	65837
72	p1	(A)10	10	65950	65959
73	p4	(AATG)3	12	66622	66633
74	p3	(TAT)4	12	67863	67874
75	p1	(A)12	12	67884	67895
76	p5	(TTTTA)3	15	68093	68107
77	p4	(GAAT)3	12	68797	68808
78	p1	(T)12	12	70181	70192
79	p1	(A)17	17	70523	70539
80	p1	(T)15	15	71210	71224
81	p1	(T)11	11	71535	71545
82	p1	(T)11	11	71560	71570
83	p4	(TAAC)3	12	71759	71770
84	p2	(TA)5	10	71775	71784
85	p1	(T)13	13	72086	72098
86	p1	(G)15	15	72218	72232

87	p2	(TA)6	12	72831	72842
88	p3	(TAT)4	12	72913	72924
89	p1	(A)16	16	73458	73473
90	p4	(AAAT)4	16	74234	74249
91	p4	(ATAA)4	16	74315	74330
92	p1	(A)12	12	75892	75903
93	p1	(A)15	15	76406	76420
94	p1	(T)11	11	76608	76618
95	p1	(A)14	14	80007	80020
96	p1	(A)14	14	81647	81660
97	p1	(A)11	11	82944	82954
98	p1	(T)10	10	83109	83118
99	p1	(T)11	11	84763	84773
100	p1	(T)11	11	87079	87089
101	p1	(T)15	15	89560	89574
102	p2	(TA)7	14	99799	99812
103	p1	(A)11	11	101122	101132
104	p4	(TTAT)3	12	104467	104478
105	p1	(A)14	14	113511	113524
106	p1	(T)10	10	117101	117110
107	p1	(T)11	11	117856	117866
108	p1	(T)11	11	118595	118605
109	p1	(T)18	18	118712	118729
110	p1	(T)10	10	118961	118970
111	p1	(T)10	10	119009	119018
112	p1	(A)10	10	119187	119196
113	p5	(AATAA)3	15	122191	122205
114	p1	(A)10	10	122579	122588
115	p4	(TCTA)3	12	122789	122800
116	p2	(AT)5	10	122829	122838
117	p4	(TTGA)3	12	122914	122925
118	p3	(AAT)4	12	128834	128845
119	p1	(A)16	16	129152	129167
120	p1	(T)14	14	130392	130405
121	p1	(T)10	10	131549	131558
122	p1	(A)10	10	132611	132620
123	p6	(AGTATC)3	18	132810	132827
124	p3	(TTC)4	12	133902	133913
125	p1	(T)11	11	134200	134210
126	p1	(T)14	14	135879	135892
127	p4	(ATAA)3	12	144925	144936
128	p1	(T)11	11	148271	148281
129	p2	(AT)7	14	149590	149603

Table S4 Simple sequence repeats (SSRs) in *A. contorta* chloroplast genome.

SSR nr.	SSR type	SSR	size	start	end
1	p6	(CATATA)3	18	110	127
2	p1	(A)12	12	1555	1566
3	p1	(T)11	11	1857	1867
4	p1	(A)16	16	2846	2861
5	p2	(AT)5	10	3859	3868
6	p2	(TA)9	18	3961	3978
7	p3	(ATA)4	12	4409	4420
8	p2	(AT)7	14	4578	4591
9	p2	(TA)5	10	4693	4702
10	p1	(A)11	11	4715	4725
11	p1	(T)12	12	4814	4825
12	p1	(A)10	10	5202	5211
13	p1	(C)10	10	5256	5265
14	p1	(A)11	11	6792	6802
15	p1	(A)22	22	7568	7589
16	p1	(A)16	16	8331	8346
17	p1	(T)11	11	8737	8747
18	p1	(A)10	10	8798	8807
19	p1	(G)10	10	8991	9000
20	p4	(TTTA)3	12	9853	9864
21	p1	(A)14	14	10104	10117
22	p1	(A)17	17	10233	10249
23	p1	(A)11	11	12005	12015
24	p2	(TA)5	10	12782	12791
25	p3	(ATT)4	12	13000	13011
26	p3	(TTA)5	15	13460	13474
27	p2	(AT)5	10	14605	14614
28	p2	(AT)8	16	14783	14798
29	p1	(T)10	10	15217	15226
30	p1	(T)10	10	17057	17066
31	p2	(AT)5	10	20658	20667
32	p1	(T)11	11	23740	23750
33	p1	(T)10	10	26995	27004
34	p1	(T)10	10	28556	28565
35	p1	(A)10	10	28582	28591
36	p1	(A)12	12	29007	29018
37	p1	(A)10	10	29020	29029
38	p1	(A)10	10	29644	29653
39	p1	(T)11	11	29827	29837
40	p1	(T)11	11	29841	29851
41	c	(C)14(T)14	28	30575	30602
42	p1	(A)15	15	31357	31371

43	p1	(T)10	10	31575	31584
44	p1	(A)11	11	31679	31689
45	p1	(A)10	10	31775	31784
46	p2	(TA)9	18	33178	33195
47	p1	(T)10	10	34284	34293
48	c	(C)11(T)11	22	34536	34557
49	p5	(ATATA)3	15	34731	34745
50	p5	(TAATA)3	15	34756	34770
51	p4	(TTTG)3	12	35090	35101
52	p1	(T)11	11	37762	37772
53	p1	(A)10	10	38156	38165
54	p1	(A)18	18	38187	38204
55	p1	(T)15	15	38602	38616
56	p1	(A)14	14	38686	38699
57	p2	(AT)12	24	38809	38832
58	p1	(A)11	11	44926	44936
59	p1	(T)10	10	45781	45790
60	p1	(T)10	10	46757	46766
61	p1	(A)10	10	46821	46830
62	p1	(A)14	14	47089	47102
63	p3	(TTA)4	12	49428	49439
64	p2	(TA)8	16	49492	49507
65	p2	(AT)6	12	49509	49520
66	p3	(ATA)4	12	49564	49575
67	p2	(TA)9	18	49592	49609
68	p1	(A)13	13	50220	50232
69	p1	(A)11	11	50263	50273
70	p2	(TA)5	10	50474	50483
71	p1	(T)10	10	50777	50786
72	p4	(TTTG)3	12	51412	51423
73	p2	(AT)7	14	53971	53984
74	p2	(AT)10	20	53995	54014
75	p2	(AT)5	10	54098	54107
76	p1	(T)14	14	54291	54304
77	p3	(TTA)4	12	54606	54617
78	p1	(A)10	10	55302	55311
79	p1	(T)14	14	56282	56295
80	p4	(ATAC)4	16	61105	61120
81	p4	(ATTA)4	16	63256	63271
82	p2	(AT)5	10	63298	63307
83	p3	(CAA)4	12	63510	63521
84	p1	(A)13	13	64015	64027
85	p2	(AT)7	14	64036	64049
86	p1	(T)12	12	65351	65362

87	p1	(A)13	13	65784	65796
88	p1	(A)10	10	65907	65916
89	p2	(TC)5	10	65969	65978
90	p4	(AATG)3	12	66579	66590
91	p3	(TAT)4	12	67825	67836
92	p1	(A)13	13	67858	67870
93	p4	(GAAT)3	12	68794	68805
94	p1	(A)12	12	70154	70165
95	p1	(T)10	10	70182	70191
96	p1	(A)14	14	70538	70551
97	p1	(T)10	10	71056	71065
98	p1	(T)11	11	71214	71224
99	p4	(TAAC)3	12	71735	71746
100	p2	(TA)11	22	71751	71772
101	p1	(T)13	13	72065	72077
102	p1	(G)15	15	72192	72206
103	p4	(ATAA)3	12	72707	72718
104	p2	(TA)7	14	72803	72816
105	p2	(TA)9	18	72820	72837
106	p6	(TTCTAT)3	18	72851	72868
107	p1	(A)12	12	73458	73469
108	p3	(ATA)4	12	73561	73572
109	p1	(T)14	14	73601	73614
110	p1	(A)12	12	74292	74303
111	p4	(ATAA)3	12	74374	74385
112	p1	(A)17	17	76476	76492
113	p1	(T)12	12	76680	76691
114	p2	(TA)5	10	76799	76808
115	p1	(A)13	13	80123	80135
116	p1	(A)19	19	81771	81789
117	p1	(A)21	21	83068	83088
118	p1	(T)10	10	84907	84916
119	p1	(A)10	10	86518	86527
120	p1	(T)13	13	87245	87257
121	p1	(T)14	14	89738	89751
122	p2	(TA)5	10	99982	99991
123	p1	(A)10	10	101301	101310
124	p4	(TTAT)3	12	104657	104668
125	p1	(T)10	10	108695	108704
126	p1	(A)15	15	113707	113721
127	p1	(A)10	10	115310	115319
128	p1	(T)10	10	117560	117569
129	p1	(T)11	11	118322	118332
130	p3	(TAT)4	12	118540	118551

131	p1	(T)12	12	119078	119089
132	c	(TG)5(T)11	21	119189	119209
133	p5	(ACATA)3	15	119344	119358
134	p1	(T)11	11	119419	119429
135	p1	(T)10	10	119472	119481
136	p1	(A)12	12	119649	119660
137	p5	(AATAA)3	15	122667	122681
138	p1	(A)10	10	123055	123064
139	p4	(TCTA)3	12	123273	123284
140	p2	(AT)5	10	123299	123308
141	p4	(TTGA)3	12	123377	123388
142	p1	(T)11	11	126240	126250
143	p3	(AAT)6	18	129279	129296
144	p1	(T)13	13	130827	130839
145	p1	(T)10	10	132043	132052
146	p1	(A)10	10	133099	133108
147	p3	(TTC)4	12	134390	134401
148	p1	(T)14	14	134694	134707
149	p1	(T)15	15	136637	136651
150	p1	(A)10	10	141654	141663
151	p4	(ATAA)3	12	145690	145701
152	p1	(T)10	10	149048	149057
153	p2	(AT)5	10	150366	150375

Table S5 Primer sequences at the boundaries between single copy and IR regions.

Species	Regions	Forward/Reverse	Primer sequence (5' to 3')
A. debilis	LSC-IRa	F	TTTGGTTGCTAGTCCCCCAC
		R	AGGCACGGCCATACATAACA
	IRa-SSC	F	TAGTGGCTCCGTTTCATGCC
		R	GCGTACCTTCTATATGGTTCCGTT
	SSC-IRb	F	GTGACCGATTAACCAACCGAC
		R	GTAGTGGCTCCGTTTCATGC
	IRb-LSC	F	CCTTAGGCACGGCCATACA
		R	ACTTCCCTCTAGACCTGGCTG
A. contorta	LSC-IRa	F	TGGTTGCTAGTCCCCCACTT
		R	AGGCACGGCCATACATAACA
	IRa-SSC	F	TAGTGGCTCCGTTTCATGCC
		R	TGTCAAAGTGGGTCCTAAGAGA
	SSC-IRb	F	AATAGCAGCCCAATCCATTTCA
		R	GCTCCGTTTCATGCCTCATTT
	IRb-LSC	F	CCGATATGCCCTTAGGCACG
		R	CAACCGTGCAAACCTTGGTA

Table S6 GenBank accession numbers of dicots with complete chloroplast genome sequences used for phylogenetic analyses.

GenBank acc.	Species	GenBank acc.	Species
NC_034692	*Piper nigrum*	NC_022457	*Berberis bealei*
NC_027941	*Piper kadsura*	NC_004993	*Calycanthus floridus var. glaucus*
NC_034697	*Piper auritum*	NC_029427	*Akebia trifoliata*
NC_008457	*Piper cenocladum*	NC_034700	*Laurus nobilis*
NC_009598	*Chloranthus spicatus*	NC_034926	*Phoebe bournei*
NC_026565	*Chloranthus japonicus*	NC_028073	*Machilus yunnanensis*
NC_020316	*Magnolia officinalis*	NC_034908	*Schisandra chinensis*
NC_020318	*Magnolia grandiflora*	NC_031849	*Trollius chinensis*
NC_030504	*Liriodendron chinense*	NC_008796	*Ranunculus macranthus*
NC_008326	*Liriodendron tulipifera*	NC_028000	*Clematis terniflora*
NC_029432	*Stephania japonica*	NC_030761	*Aconitum carmichaelii*
NC_030166	*Annona cherimola*	NC_034347	*Fragaria pentaphylla*
NC_029428	*Epimedium sagittatum*	NC_032038	*Rosa roxburghii*
NC_029943	*Epimedium koreanum*	NC_022868	*Glycine soja*
NC_008336	*Nandina domestica*	NC_018051	*Vigna unguiculata*
KJ468103	*Tamarindus indica*	NC_006290	*Panax ginseng*
NC_020431	*Salvia miltiorrhiza*	NC_001879	*Nicotiana tabacum*
NC_001879	*Arabidopsis thaliana*		
